# Bibliometric and Visualization Analysis of the Ecology of Men’s Sexual and Reproductive Healthcare Research in MENA (1985–2022): Outputs, Trends, Shortcomings and Hotspots

**DOI:** 10.1080/2090598X.2022.2141864

**Published:** 2022-11-04

**Authors:** Walid El Ansari, Mohamed Arafa, Ahmad Majzoub, Haitham Elbardisi, Ahmed Albakr, Mohammed Mahdi, Kareem El-Ansari, Abdulla Al Ansari, Khalid AlRumaihi

**Affiliations:** a Department of Surgery, Hamad Medical Corporation, Doha, Qatar; b College of Medicine, Qatar University, Doha, Qatar; c Weill Cornell Medicine – Qatar, Doha, Qatar; d Urology Department, Hamad Medical Corporation, Doha, Qatar; e Andrology Department, Cairo University, Cairo, Egypt; f Faculty of Medicine, Ain Shams University, Cairo, Egypt

**Keywords:** Bibliometric, MENA, men, sexual, reproductive, healthcare, Arab

## Abstract

**Background:**

To date, no previous research assessed the bibliometrics of men’s sexual and reproductive healthcare (SRHC) across Arab countries. This study appraised the current standing of men’s SRHC research in the MENA (Middle East and North Africa) region.

**Methods:**

We performed a bibliometric analysis to assess qualitatively and quantitatively the peer-reviewed articles published from Arab countries from inception to 2022. In addition, we conducted a visualization analysis, and assessed outputs, trends, shortcomings and hotspots over the given time period.

**Results:**

There was a generally low numbers of publications, 98 studies were identified, all with cross-sectional design, and two thirds explored prevention and control of HIV/other STDs. Studies were published in 71 journals, of which the Eastern Mediterranean Health Journal, Journal of Egyptian Public Health Association, AIDS Care and BMC public health were most common. The Journal of Adolescent Health, Fertility Sterility and Journal of Cancer Survivorship were among the highest IF ranking. Publishers were commonly USA or UK-based, median journal IF was 2.09, and five articles were in journals of IF > 4. Saudi Arabia had the highest published output followed by Egypt, Jordan and Lebanon, while 10 Arab countries had no publications on the topic. Corresponding authors expertise fields were most commonly public health, infectious diseases and family medicine). Collaborations in-between MENA countries were notably low.

**Conclusions:**

There is general paucity of published outputs on SRHC. More research across MENA is needed, with more inter-MENA collaborations, and with inclusion of countries that currently have no outputs on SRHC. In order to accomplish such goals, R&D funding and capacity building are required. Research and published outputs should address SRHC burdens.

## Introduction

Despite the positive relationship between male reproductive health and overall health, men are generally less likely to engage with sexual and reproductive healthcare (SRHC)[1]. Globally, few men with sexual dysfunction seek healthcare [2,3]; young men require additional education and knowledge about SRHC access and fertility awareness [4,5]; and low male involvement is a barrier to family planning[6]. Likewise, across the Arab countries, men were more likely to hide their sexual health impairment rather than consult a physician[7].

Men’s limited engagement with SRHC could be due to several reasons. Sexual health is shaped by cultural, economic, religion, traditions and the social construction of gender roles/ sexual behaviours in society[8]. These might view men as independent/ invulnerable, and with a higher threshold of accepting SRH problems[9]. Likewise, in the Middle East, taboos associated with sexual health might prevent men from seeking healthcare[8]. Men’s suboptimal engagement with SRHC might also be due to a lack of response of the health system or healthcare organisation and delivery to men’s needs[10]; lack of support or guidelines for health professionals to promote men’s SRH[11]; or men’s lack of knowledge of SRHC services. Compared to same-age college women, men were much less knowledgeable about SRHC services access[4]; and SRH outcomes generally emphasize women/girls, over men[12]. Likewise, evidence suggests the need for family planning programs targeting men[13], and gender-specific messages to increase men’s engagement with SRH services[4]. Regardless of whether the reason is gender- or health service-related, the outcome remains the same: a decreased likelihood of men’s engagement with SRHC.

To date, the current state of men’s SRHC across Arab countries has not been well-characterized, despite the cultural sensitivities and taboos surrounding sexuality in MENA[8]. Some authors maintain that even with the progress achieved in the availability and provision of SRHC services across Arab countries, efforts are required to ensure that needs are met[7]. Thus, taking stock of the current literature pertaining to men’s SRHC is key to recognize potential service- and research-related gaps and opportunities, as well as to guide future funding priorities[14].

Bibliometric analysis is a useful tool as it allows identification, classification and comparison of information about the current state of affairs in a given subject[15]. Bibliometric analyses of the scientific literature is frequently employed to explore and evaluate practice and research in urology, with topics ranging from COVID-19 and urology (Soytas et al 2021)[16], to appraising urology/nephrology research activity across a region[17], or gauging a country’s contribution to urology/nephrology research[18]. Such analyses of the published literature can also guide national scientific agendas and provide data on return on research [19,20].

Given the knowledge gap highlighted above, the current study appraised, by country, the published outputs concerning men’s SRHC. The specific objectives were to analyze the contribution of the Arab scientific community in terms of (i) men’s SRHC literature; (ii) regional/international collaborative patterns; (iii) productivity across institutions; (iv) characteristics of highly cited papers; (v) characteristics of corresponding authors; and (vi) research outputs, trends, shortcomings and hotspots. The findings provide the research growth in the field of men’s SRHC, to assist researchers and practitioners. We aimed to provide recommendations for active steps to improve the current situation based on our findings.

## Materials and methods

### Settings

The Arab nations cover a large geographic area including North Africa, parts of Asia and the Arabic Peninsula with about 500 million inhabitants, of which slightly more than half (51.82%) are males[21]. Reports confirm the lack of data pertaining to men’s SRH in the MENA region; the lack of knowledge and poor attitude regarding screening and treatment as challenges for men’s RH in the MENA region; and that some Arab nations have implemented health system reforms, but further efforts are still needed for the full integration of SRH within current primary health care in the Arab countries[22]. Despite such state of affairs, to the best of our knowledge, there are no published reports covering the Arab world specifically about the bibliometrics of SRHC for men. Hence, the findings of the current study will serve as a useful approach to benchmark, collate and compare scientific output on the topic across the Arab world, appraise practice, identify knowledge gaps and guide the future planning of SRHC as well as allocation research spending.

### Definitions

Sexual health is defined as the Integration of the somatic, emotional, intellectual, and social aspects of sexual being, in ways that are positively enriching and that enhance personality, communication, and love[23]. Reproductive health is a state of complete physical, mental and social well-being and not merely the absence of disease or infirmity, in all matters relating to the reproductive system and to its functions and processes[24]. MENA countries include Algeria, Bahrain, Djibouti, Egypt, Iraq, Jordan, Kuwait, Lebanon, Libya, Morocco, Oman, Qatar, Saudi Arabia, Syria, Tunisia, United Arab Emirates, Palestine, Yemen, Sudan, Mauritania, Western Sahara.

### Sources of data and searching strategies

A structured literature search was done using electronic databases that included PubMed and WoS across the timespan from initiation of the databases till June 30^th^, 2022. Search was limited to English language using specific search MeSH (online supplemental Box 1).

### Eligibility criteria and Study Selection

The inclusion criteria were public health manuscripts discussing men’s SRH in MENA region. Clinical articles, editorials, reviews, letters, congress proceedings, books and duplicates were excluded. Manuscripts dealing with women SRH or from countries other than MENA were also excluded. All eligible articles were screened by 2 reviewers (MA, WEA) working simultaneously together. The reviewers evaluated the titles for eligibility, and in cases of disagreement, consensus was reached through discussion.

### Data retrieval

Raw data was extracted into a workbook on Excel 2016 (Redmond, WA, USA). The data included; 1) journal information [country of origin, ranking (Q), H-index, impact factor (IF) and citescore]; 2) manuscript information (number of authors, collaborative institutes/countries and country of correspondence); and, 3) corresponding author information (name, institute, H-index, number of citations).

### Ethics

The Medical Research Center (IRB- Institutional Review Board) at Hamad Medical Corporation does not require submission of an IRB application for this type study as there is no risk for human subjects in such publications given the data are based on public documents and published literature and did not involve any interactions with human subjects.

## Analysis

In line with others, we aimed to analyze the published outputs from the Arab countries quantitatively and qualitatively by examining publication count (quantitative) and through the use of journal IF and citation counts (as quality indicators)[25].

### Statistical Analysis

The skewness and kurtosis tests were used for testing the normal distribution of continuous variables. Frequencies and percentages summarized categorical variables. Chi squared $\chi^{2}$ test (or Fisher’s exact test, as appropriate) was used for categorical data while one-way ANOVA test was used for continuous variables normally distributed. Kruskal-Wallis H test was used for continuous variables that were not normally distributed.

We reviewed the characteristics of publications by establishing ‘The WoS Literature Analysis Report’ online, including distribution of countries, institutions, journals and authors, number of annual publications, citation counts, and H-index. The ‘H-index’ is a recognized reliable method of forecasting future research, and includes time-cited publications of a given country compared to the number of times for which those publications are at least co-cited.[26] A comparison of number of publications, citations, and journal/author H-indices across the Arab countries was undertaken using GraphPad Prism 6. The statistical results were then displayed using CiteSpace. The consequence and number of co-cited authors and co-cited references were calculated via VOSviewer (Leiden University, Leiden, Netherlands). Network were generated for co-authorship and MeSH keywords using the VOS viewer software (freely available at https://vosviewer.com), and heat maps were utilized to illustrate the density of publications by country.

In addition, in order to qualitatively analyze the retrieved articles, we employed the WHO’s Framework for operationalising sexual health and its linkages to reproductive health[23]. The publications emerging from each country were categorized based on the framework in order to outline the different domains of men’s SRHC that each country was attempting to target. Based on WHO’s (2017) categorization, 8 SRH domains are identified, comprising: Antenatal, intrapartum & postnatal care; Comprehensive education & information; Contraception counselling & provision; Gender-based violence prevention, support & care; Fertility care; Prevention/control of HIV & other sexually transmissible infections; Safe abortion care; and Sexual function & psychosexual counselling[23].

## Results

### Identified studies

**Figure 1.**
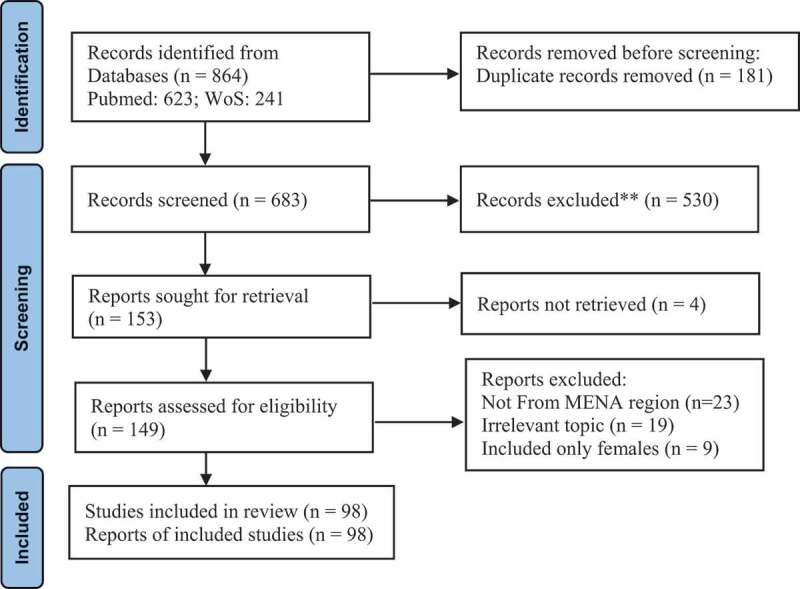
PRISMA flow chart of search results of men’s experiences in sexual and reproductive healthcare in MENA countries.

Figure 1. PRISMA (Preferred Reporting Items for Systematic Reviews and Meta-Analyses) flow chart of search results of men’s experiences in sexual and reproductive healthcare in MENA countries.

### Characteristics of studies and topics

A total of 98 studies met the inclusion criteria and were included in the analysis. The complete list of retrieved articles, corresponding authors, their affiliations and publishing journals is presented in Supplementary Table 1. All articles had an original cross-sectional design.

**Figure 2.**
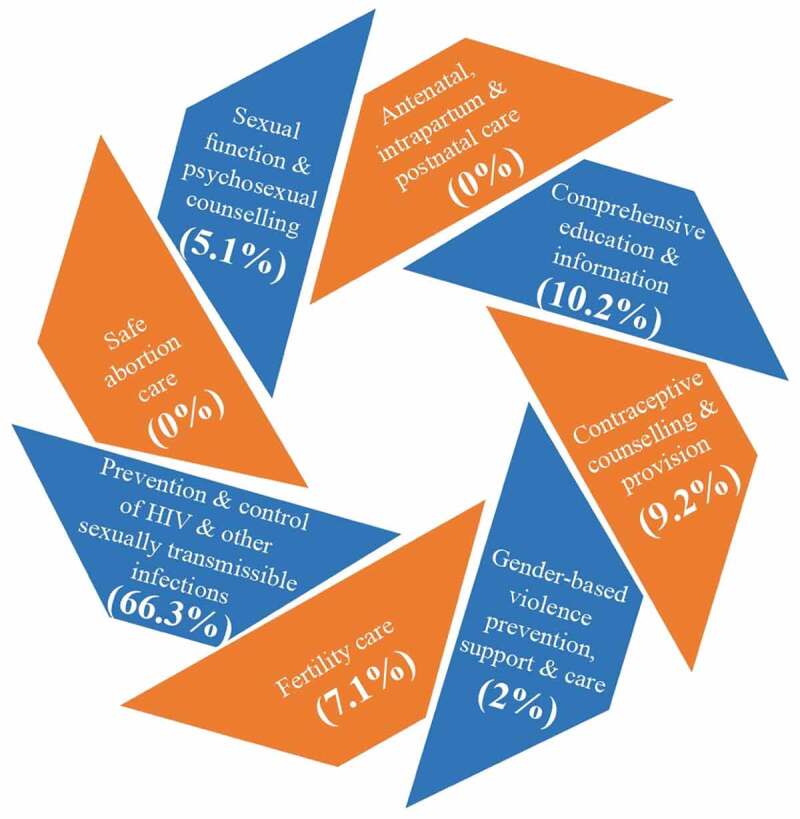
Description of included studies using the WHO framework (WHO 2017).[23]

Figure 2 shows the description of included studies using the WHO framework[23].

Two third of the studies (66.3%) explored prevention and control of HIV and/or other STDs. Among the remaining one third, comprehensive education and information and contraceptive counseling and provision were the most commonly discussed topics. There was a strong focus on HIV/AIDS; weak focus on fertility/sexual dysfunctions; no publications on men’s involvement in sexual gender-based violence; no studies of men’s involvement in antenatal/intrapartum/postnatal care; and, many studies identifying lack of SRH knowledge, but no publications on policies and strategies addressing such shortcoming.

**Figure 3.**
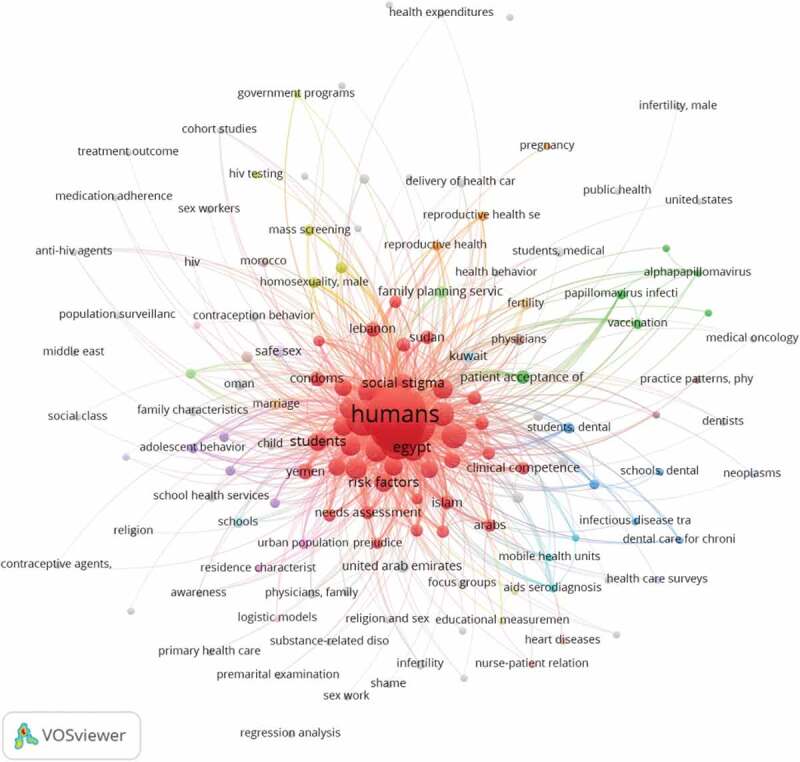
Network visualization of most frequent MeSH Keywords of retrieved studies.

**Figure 4.**
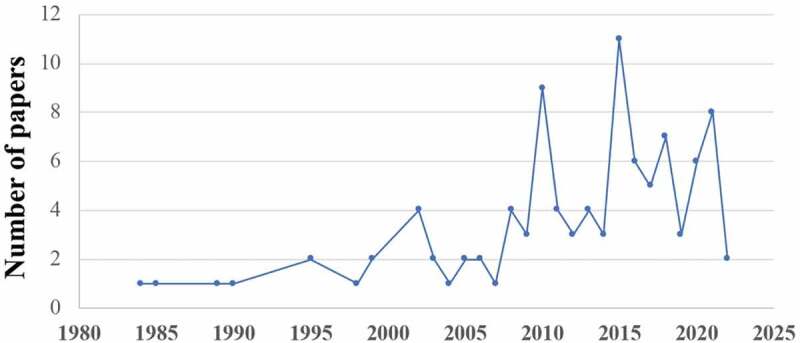
Growth of men’s SRHC published outputs in Arab countries: Number of publications by year.

Mapping the most frequent MESH keywords across all documents revealed 13 clusters comprising 64 terms that had an occurrence of >4 times among the included studies (Figure 3). Figure 4 shows that the first study was published in 1984, and more than half the studies were published during the past decade.

### Journal characteristics and metrics

In the current study, we identified 71 academic journals that published topics related to the keywords selected in this review (Supplementary Table 1). The most commonly publishing journals were Eastern Mediterranean Health Journal (10 papers [10.2%], IF 2022 = 1.628), Journal of Egyptian Public Health Association (5 papers [3.1%], IF 2022 = 1.444), AIDS Care (4 papers [4.1%], IF 2022 = 2.32) and BMC public health (3 papers [3.1%], IF 2022 = 3.98). As for IF, The Journal of Adolescent Health (1 paper [1.1%], IF 2022 = 5.012], Fertility Sterility (1 paper [1.1%], IF 2022 = 4.72) and Journal of cancer survivorship (1 paper [1.1%], IF 2022 = 4.442) were among the highest-ranking publishing journals.

### Geographic characteristics

**Figure 5.**
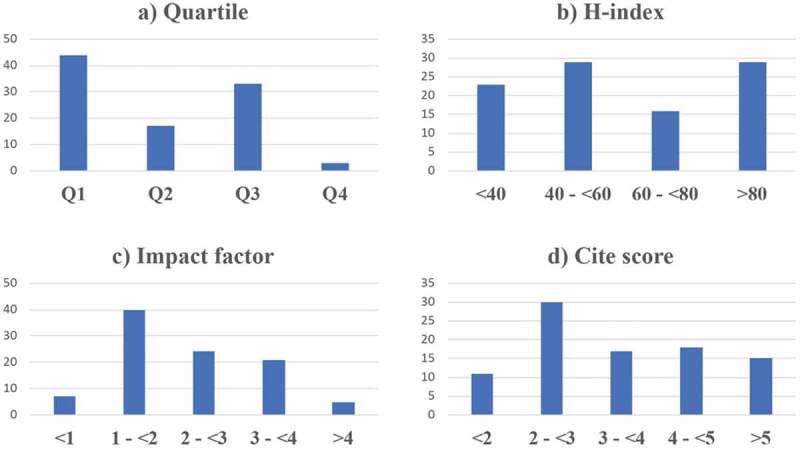
Journal metrics: Quartiles, H-index, impact factor and cite score of publishing journals.

When the retrieved data were analyzed by publisher and journal, articles were most commonly issued by publishers based in the USA followed by UK, Switzerland, Netherlands among others. The median (IQR) H-index, impact factor and cite score of the publishing journals were 55 (40.5–101), 2.09 (1.39–3.02) and 3.3 (2.3–4.5), respectively. More than half of the journals belonged to the first or second quartile of their respective field of study, and 46.4% of them had an H-index >60 (Figure 5 a and b). Five articles were published by journals having an impact factor >4, while the majority of the remaining articles (n = 85) were published by journals whose impact factor was >1 (Figure 5c). A journal cite score of 2 to <3 was observed in 30 publications. An almost equal number of articles were published in journals with cite score of 3 to <4, 4 to <5, and >5 (Figure 5d).

When the data were analyzed by country, Saudi Arabia (20, 20.4%) had the highest published output followed by Egypt (18, 18.4%), Jordan (7, 7.1%) and Lebanon (7, 7.1%). No publications related to men’s SRHC was found from Algeria, Bahrain, Libya, Palestine, Qatar, Syria, Tunisia, Djibouti, Mauritania or Western Sahara. Non-Arab countries whose researchers collaborated most with researchers in the Arab world included USA (8, 8.1%) and Netherlands (4, 4.1%). Table 1 shows the distribution of publications by country categorized by WHO men’s sexual and reproductive healthcare domains.

**Table 1.** Publications by country categorized by WHO men’s sexual and reproductive healthcare domains.

Country	Domain of men’s sexual and reproductive healthcare							
	1	2	3	4	5	6	7	8
Saudi Arabia		✓	✓		✓	✓✓✓		✓
Egypt		✓		✓	✓	✓✓✓		
Lebanon		✓		✓	✓	✓		
Jordan		✓	✓			✓		✓
Kuwait						✓✓		
Oman						✓✓		
United Arab Emirates			✓			✓		
Sudan						✓		
Yemen						✓		
Morocco					✓	✓		
Iraq						✓		
Bahrain, Algeria, Libya, Palestine, Qatar, Syria, Tunisia, Djibouti, Mauritania, Western Sahara								

Cell values reflect number of publications of a given country addressing the given SRH domain (**✓ **= 1–3 publications; **✓✓ =** 4–6 publications; **✓✓✓ **= >6 publications). SRH sexual and reproductive healthcare (SRH) Domains: 1. Antenatal, intrapartum & postnatal care; 2. Comprehensive education & information; 3. Contraception counselling &provision; 4. Gender-based violence prevention, support & care; 5. Fertility care; 6. Prevention/control of HIV & other sexually transmissible infections; 7. Safe abortion care; 8. Sexual function & psychosexual counselling (based on WHO 2017, ‘Sexual health and its linkages to reproductive health: an operational approach’).

### Author characteristics

**Figure 6.**
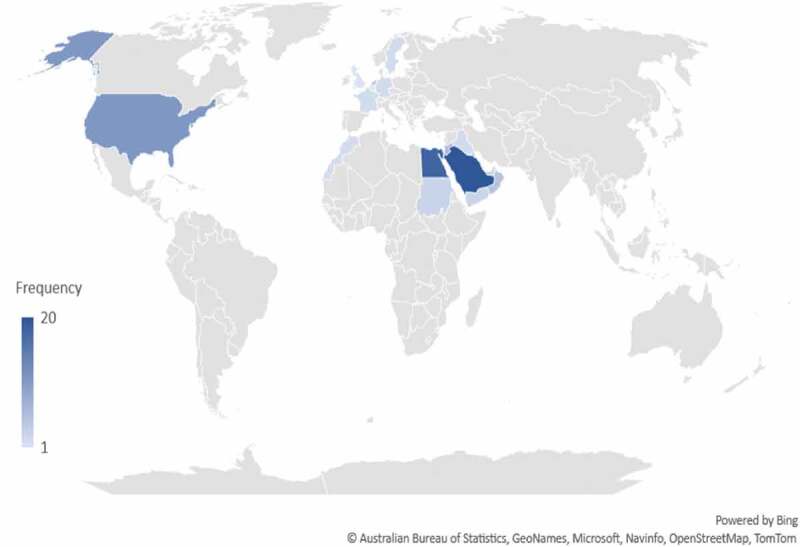
Distribution of corresponding authors’ origins.

**Figure 7.**
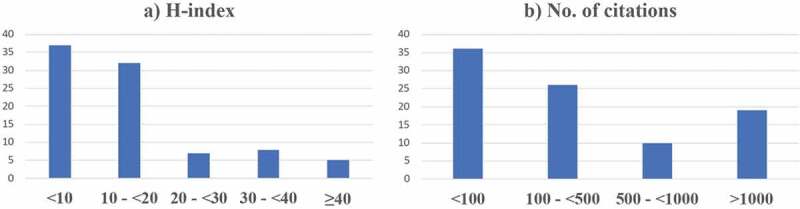
Author metrics: H-index and number of citations by author.

**Figure 8.**
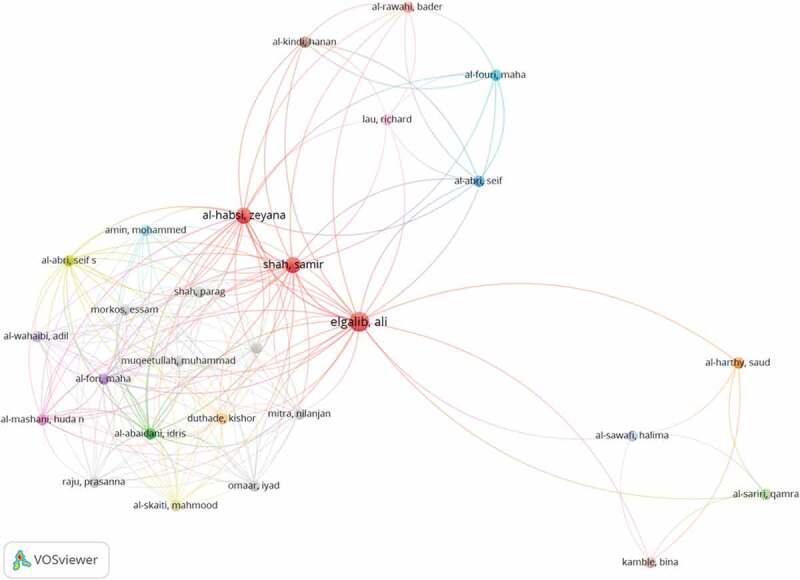
Network visualization of level of cooperation among identified authors.

The majority of the affiliations of the corresponding author were from Arab countries (n = 78), and most of the publications were collaborations with other Arab or non-Arab countries (n = 63). Figure 4 depicts the countries of origin for the corresponding authors. Public health, infectious diseases and family medicine were the 3 most common fields of expertise for the corresponding authors (Figure 6). Median (IQR), H-index and number of citations for the corresponding authors were 10 (5.75–19) and 176.5 (26.25–894.5), respectively. Most authors had H-index <20 (n = 69) and <500 citations (Figure 7). Figure 8 maps the extent of cooperation among the identified authors of the retrieved studies; and Table 2 presents the top 10 ranking of authors who published in the men’s SRHC field from the Arab world, and their affiliations.

**Table 2.** Ranking of top 10 corresponding authors published in men’s SRHC field across 21 Arab countries, or who collaborated with authors located in Arab countries.

SCR	Author	H-index	Affiliation*
1	Ziad A. Memish	128	Al Faisal University
2	Marcia C. Inhorn	56	Yale University
3	Laurence Weiss	51	Hôpital Européen Georges-Pompidou
4	Patrizia Carrieri	49	Aix Marseille University
5	Pascale Salameh	43	Lebanese University
6	Ali A. AL-Jabri	39	Sultan Qaboos University
7	Mark Hayter	38	King Abdullah University
8	Ahmed Mahfouz	34	King Saud University
9	Mostafa A. Abolfotoh	33	King Saud University
10	Yagob Y Al Mazrou	33	Ministry of Health, KSA

As shown in ISI web of knowledge last publication; SCR: standard competition ranking.

### Institution characteristics

Table 3 shows the list of active institutions with >1 publication in the field of men’s SRHC in Arab countries.

**Table 3.** Active institutions with more than 1 publication in men’s SRHC in the 21 Arab countries.

Institute	Country	Value
1 King Saud University	KSA	7 (7.1)
2 University of Tanta	Egypt	4 (4.1)
3 Kuwait University	Kuwait	4 (4.1)
4 University of Science and Technology	Jordan	4 (4.1)
5 Maastricht University	Netherlands	4 (4.1)
6 Alexandria University	Egypt	3 (3.1)
7 University of Sanaa	Yemen	3 (3.1)
8 King Abdulaziz University	KSA	3 (3.1)
9 American University of Beirut	Lebanon	2 (2)
10 United Arab Emirates University	UAE	2 (2)
11 Sultan Qaboos University	Oman	2 (2)
12 King Khalid University	KSA	2 (2)
13 University of Jordan	Jordan	2 (2)
14 Hashemite University	Jordan	2 (2)
15 Suez Canal University	Egypt	2 (2)
16 Harvard TH Chan School of Public Health	USA	2 (2)
17 Hawler Medical University	USA	2 (2)

Cell values indicate number of publications (%) **SCR** standard competition ranking, equal institutions have the same ranking number, and then a gap is left in the ranking numbers.

## Discussion

Bibliometric analysis can identify, classify and compare information about the current state of a given subject, or gauge individual countries’ contributions to the subject [15,18]. There is a lack of bibliometric studies regarding men’s SRHC in Arab world that evaluates the research output in a qualitative and quantitative manner. The current study is the first comprehensive bibliometric appraisal of men’s SRHC research across the 21 MENA nations characterizing the published articles and scientific research output of the Arab world pertaining to men’s SRHC across the last 37 years.

Our main findings were that 98 studies were identified, all with cross-sectional design, and two thirds (66.3%) explored prevention and control of HIV and/or other STDs. Studies were published in 71 journals, of which the Eastern Mediterranean Health Journal, Journal of Egyptian Public Health Association, AIDS Care and BMC public health were most common. Median journal IF was 2.09, five articles were in journals of IF > 4[26]. The Journal of Adolescent Health, Fertility Sterility and Journal of Cancer Survivorship were among the highest IF ranking journals, and publishers were most commonly USA or UK-based. KSA had the highest published output followed by Egypt, Jordan and Lebanon, while 10 countries had no publications on the topic. Public health, infectious diseases and family medicine were the most common expertise fields of the corresponding authors. Collectively, the present bibliometric analysis revealed several trends, shortcomings, strengths, hotspots and mismatches.

In terms of trends, we observed a number of shortcomings that included: 1) Generally low numbers of publications (N = 98 across 35 years), and almost half the MENA countries had no publications on men’s SRHC during the period under examination; 2) Men’s SRHC studies in MENA undertaken by authors from outside MENA were not uncommon; 3) Collaborations in-between MENA countries were notably low i.e. when cross-country collaboration was identified, it was usually with a non-MENA country, most commonly USA; and, 4) Females were underrepresented among the corresponding authors.

We also observed that: 1) Starting with low numbers, publications on men’s SRHC from MENA showed a healthy increase over the past decade or so, where generally, from 2010 onwards, the number of annual publications nearly doubled compared to pre-2010; 2) Many of the top-ranking authors identified had notable H indices, ranging from 33 to 128, with half of them exhibiting H indices >42; and, 3) Journals where articles were published included many with high IF, ranging from 2.32 to 5.0, with a majority being around IF 2.

Regarding noteworthy hotspots and mismatches, the current analysis discovered a strong focus on HIV/AIDS, when MENA has low HIV prevalence; weak focus on fertility/sexual dysfunctions, despite their high prevalence in MENA; no publications on men’s involvement in sexual gender-based violence, despite its frequency across MENA; no studies of men’s involvement in antenatal/intrapartum/postnatal care, despite the international literature valuing such involvement; and, many studies identifying lack of SRH knowledge, but no publications on policies and strategies addressing such shortcoming (Figure 2 and Table 2). Below we discuss each in turn.

In terms of trends and shortcomings, we noted that the number of publications on men’s SRHC were low, reflecting a generally low trend of research publications on other topics from the Arab world. For instance, a recent review of published economic evaluation studies from MENA observed very low output[27]; and similarly, pancreatic cancer and breast cancer bibliometric reviews also confirm the low contribution of Arab countries [28,29]. Whilst the precise reasons for such low output could be numerous, some authors proposed that it might reflect the regional instabilities and civil wars in MENA that impede medical research, increase brain drain, lead to isolation of research groups, and reallocate medical research funds towards other domains and to humanitarian relief [30,31]. In addition, research activity and capacity of any given country is subject to factors that include national income and population size[17]. Other factors include the amount of R&D funding allocated, the prevailing competitive atmosphere amongst institutes and individuals, and the dominant education system in the country that can significantly impact research productivity, as well as whether residency programmes have integrated research rotations in their curricula, which influences research outcomes [17,30,32]. Notwithstanding, a point to be considered is that identified publications might not correctly reflect research efforts on men’s SRHC across MENA, where studies are more likely to be written in local languages for local authorities, limiting their chances of publication, as noted by others[27].

In terms of growth, our analysis found that MENA has been experiencing a slow but steady growth in men’s SRHC research output since 1984. Such growth concurs with reports that the annual number of research documents published from Arab countries is increasing. For example, there was a steady growth in pancreatic cancer research output since the beginning of the century[28]; and obesity research from MENA remained low until the mid-1990s but exhibited a steady increase after the year 2000[17]. Such trends are also seen in men’s health research globally, for example, in premature ejaculation research[33].

However, our analysis suggested that such growth has been limited to a few countries (e.g. KSA and Egypt), concurring with others[28]. Indeed, we found that four countries (KSA, Egypt, Jordan, Lebanon) contributed 53% of the SRHC publications, while almost half the MENA countries had no publications on the topic during the 37 years under examination. These findings are congruent with a scoping review of men’s SRHC in Nordic countries, where the absolute majority of the studies were conducted in Sweden, with less from Denmark and Norway, and no studies from Iceland or Greenland[34]. Notably, MENA conflict-affected countries, for example, Iraq and Yemen had one publication each and Libya, Palestine and Syria had none during the study period; and some lower-GDP countries e.g. Djibouti, Mauritania, Western Sahara had no publications on the topic. However, it was surprising that Arab nations with higher GDP, for example, Qatar and Bahrain had no publications on men’s SRHC. Perhaps this reflects a need for an increased focus on men’s SRHC as emphasized by recent WHO country reports that highlight the need for enabling environment, health systems, service delivery pertaining to SRHC in these countries [35,36].

As for countries, the high contributions from KSA followed by Egypt concurs with the Arab region’s contribution to COVID-19 research, where the largest publications volume was from KSA followed by Egypt [37,38]; and also supports obesity publications across the Arab world, where KSA had the highest quantity of documents followed by Egypt[17]. An important point is that the prolificity of research output is subject to the topic’s priority for each country, hence for example, in terms of leishmaniasis research, a bibliometric study observed that Tunisia had the greatest output from Arab world followed by Sudan[39].

Pertaining to collaborations, the current analysis found that intra-MENA collaborations were generally low, congruent with a pancreatic cancer bibliometric review where most MENA-based studies did not involve collaborations[28]. Others reported that international collaborations of, for example, Saudi Arabia in COVID-19 research output were mainly with researchers from Egypt, followed by the USA[38]; and for obesity research across Arab nations, collaborations were mostly with the USA, France and England[17]. We observed that about one third of the top 10 ranking corresponding authors published in men’s SRHC field were affiliated with institutions outside the Arab world (e.g. France or the USA). International collaboration could enhance the quantity/quality of research[40], increase the visibility of scientific publications from a given country[41], and help capacity building and making national problems more internationally observable[42]. Notwithstanding, more inter-Arab collaboration in research activity would be certainly welcomed.

In terms of gender, our observation of the low number of females as corresponding authors resonates with the underrepresentation of the female sex in medical research in MENA, despite the worldwide trend towards increasing their involvement[28].

As for mismatches, the strong HIV/AIDS focus across the published literature was despite its low prevalence in MENA (0.1%) [43,44]. Nevertheless, MENA’s political turmoil and wars might increase the vulnerability to HIV[45], reflected by the increasing HIV prevalence in MENA[46]. Likewise, our observed low focus on fertility care and sexual dysfunctions/counselling, although incongruent with the high prevalence of fertility/sexual problems in MENA (22.6%)[47], is probably due to multi-layered cultural, religious, community gender and social norms characteristic of MENA populations [48,49]. Our findings support a review of men’s SRH in Scandinavia, where sexual functioning/counselling studies spanned a very small proportion of the studies identified[34]. In addition, we noted that gender-based violence prevention, support and care represented 2% of the publications, even though women’s exposure to male domination is to an extent normalized across many Arab nations [50,51], but again resonates with a Nordic review, where sexual violence publications comprised a very small minority[34]. Similarly, we found no studies related to antenatal/intrapartum/postnatal care, although men’s involvement in maternal health is critical to enhance females’ use of such services[52], yet again reflecting the limited attention to men’s own experiences and needs during their partner’s antenatal visits [53,54].

Collectively, such identified mismatches support a very recent review of published economic evaluation studies of public health interventions from 26 MENA countries that found that the research output did not reflect current and upcoming disease burden and risk factors trends in the MENA region[27]. In the face of inevitable pressures from competing for alternative interventions, countries could direct their finite health budgets to meet the priority health needs of their populations to reach fair and efficient outcomes[55].

In terms of institutions, the current analysis found that active institutions with >1 publication in men’s SRHC across the 21 Arab countries was King Saud University, in agreement with that for COVID-19 research output across MENA, King Saud University was the most productive among all institutes in terms of COVID-19-related publications at both local and regional levels[38]. KSA was also leading in obesity research, King Saud University being the first of the top 10 active institutions in obesity research from Arab countries[17].

### Limitations and strengths

The study has limitations. It comprised original articles published between 1987 and 2022 and indexed by WoS and PubMed database. Inclusion of other electronic databases, for example, Scopus and Embase could furnish a wider coverage of scientific literature. However, the WoS provides fine grain details (e.g. annual publications, author information, journal sources, country, and institution information) that other databases do not provide[56]. Conference abstracts, books, ‘grey literature’ and other types of publications were not included in the current bibliometric analysis. We inspected only English-language journals. The country of origin was based on the affiliated institution of the corresponding author and the alternative use of primary author, could have yielded different findings. To gauge research quality, we employed the easily accessible and transferable impact factor (IF), which is appropriate when comparisons are within the same research area, nevertheless is not without some disadvantages[57].

This bibliometric analysis has many strengths. To our knowledge, it is the first to focus on men’s experiences in SRHC across the Arab countries. In line with others[56], we explored trends and hotspots, as well as knowledge gaps. To guarantee an appropriate search of the published literature, we examined the search strategy used in a similar published article on men’s SRH in Nordic countries[34], and modified the search terms they used. There was no time limitation for our literature retrieval, the data downloaded from the databases covered the majority of published outputs in the subject area, and data analysis was relatively objective and comprehensive. For the qualitative analysis, the review team was trained in the completion of the screening and data characterization forms, using several articles that were randomly selected from the literature. Data extraction of each citation and article was undertaken by two independent members of the review team who met regularly to agree on any conflicts. Synthesis of the emerging findings was guided by the WHO framework[23]. The data of our study will serve as a baseline data for evaluation of future research activities and for comparative purposes with other non-Arab countries.

## Conclusion

The prevalent general paucity of published outputs reflects a certainly urgent need for SRHC research across MENA. Inter-MENA collaborations are required as these countries significantly overlap in religion, customs and culture. Political will and leadership, as well as financial commitment, are central to SRHC research and services. Research could include the domains people-centred approaches, health systems, supportive enabling environments, and behaviour-change. Notably, quality research will need to include capacity building, and should address current and upcoming SRHC burdens.

## Supplementary Material

Supplemental Material
