# Supplementary material for: Bibliometric and Visualization Analysis of the Ecology of Men’s Sexual and Reproductive Healthcare Research in MENA (1985–2022): Outputs, Trends, Shortcomings and Hotspots
Source: Arab J Urol. 2022 Nov 4;21(2):82–93. doi: 10.1080/2090598X.2022.2141864 (PMC10208131; doi:10.1080/2090598X.2022.2141864)
Supplement: Supplemental Material [file TAJU_A_2141864_SM4602.docx]

**Supplementary Box 1: Search terms used in the current scoping review**

| (Sexual Health[mesh] OR "Sexual health" OR Reproductive Health[mesh] OR "Reproductive health" OR "Sexual and reproductive health" OR "Sexual and reproductive health and rights" OR SRHR OR "sexual function" OR "sexual functions" OR "sexual dysfunction" OR "sexual dysfunctions" OR erectile dysfunction[mesh] OR "erectile dysfunction" OR sexual satisfaction OR sex offenses[mesh] OR sexual violence OR gender-based violence[mesh] OR gender-based violence OR family planning services[mesh] OR "Family Planning" OR contraceptive agents[mesh] OR contraceptive devices[mesh] OR "contraceptive" OR "contraceptives" OR Condom[mesh] OR condom OR condoms OR Infertility[mesh] OR infertility OR fertility[mesh] OR fertility OR prostatic neoplasms[mesh] OR "Prostate cancer" OR Genital Diseases, Male[mesh] OR sexually transmitted diseases[mesh] OR "Sexually transmitted infections" OR STIs OR "Sexually transmitted diseases" OR STDs OR chlamydia[mesh] OR Chlamydia OR Gonorrhoea[mesh] OR gonorrhea OR gonorrhoeae OR Syphilis[mesh] OR Syphilis OR Trichomonas Infections[mesh] OR Trichomoniasis OR Herpes Genitalis[mesh] OR "herpes genitalis"[tiab] OR Papillomavirus Infections[mesh] OR "Papillomavirus" OR Condylomata Acuminata[mesh] OR "Genital warts" OR HIV Infections[mesh]) AND (men[mesh] OR Men OR man OR men’s OR Male[mesh] OR male OR masculinity[mesh] OR masculinity OR Men's Health[mesh] OR Homosexuality, Male[mesh] OR "MSM" OR "men having sex with men"[TIAB] OR "men who have sex with men"[TIAB] OR "men who have sex with other men"[TIAB] OR ((transgender*[TIAB] OR transgender persons[MH] OR transsexual*[TIAB]) AND man) OR transman[TIAB] OR "trans men"[TIAB] OR transmen[TIAB]) AND (Health Services Accessibility[mesh] OR Health Facilities[mesh] OR community health services[mesh] OR health services[mesh] OR health services research[mesh] OR delivery of health care[mesh] OR preventive health services[mesh] OR health services needs and demand[MH] OR quality of health care[majr:noexp] OR "Health care providers" OR "Health care"[tiab] OR "Health services"[tiab] OR "Healthcare"[tiab] OR "healthcare"[tiab] OR clinic[tiab] OR hospital[tiab] OR "primary care"[tiab]) AND (Professional-Patient Relations[mesh] OR Attitude of Health Personnel[mesh] OR Patient Satisfaction[mesh] OR Healthcare Disparities[mesh] OR patient acceptance of health care[MH] OR health care evaluation mechanisms[mesh] OR "perception"[tiab] OR "perceptions"[tiab] OR "perceive"[tiab] OR "perceived"[tiab] OR "satisfaction"[tiab] OR "expectation"[tiab] OR "expectations"[tiab] OR "experience"[tiab] OR "experiences"[tiab] OR "evaluation"[tiab] OR "assessment"[tiab] OR "quality"[tiab] OR "trust"[tiab] OR "Shame"[tiab] OR "stigma"[tiab]) AND (Epidemiologic Research Design[mesh] OR Qualitative Research[mesh] OR empirical research[mesh] OR Surveys and Questionnaires[mesh] OR survey OR surveys OR questionnaires OR questionnaire OR Empirical OR Cross-Sectional Studies OR Cohort OR CaseControl OR Observational OR Registries OR analysis OR Clinical Trials OR Meta-Analysis OR "meta analysis" OR "systematic review" OR "scoping review" OR "literature review" OR "review of literature" OR Qualitative OR "Grounded Theory" OR Interviews as Topic[mesh] OR "interviews" OR "interview" OR focus groups[mesh] OR "Focus group" OR "focus groups" OR themes[tiab]) AND (Arabic Countries[mesh] OR arabian[tiab] OR Egypt[tiab] OR Egyptian[tiab] OR Libya[tiab] OR Libyan[tiab] OR Tunis[tiab] OR Tunisian[tiab] OR Algeria[tiab] OR Algerian[tiab] OR Morocco[tiab] OR Moroccan[tiab] OR Sudan[tiab]OR Sudanese[tiab] OR Jordan[tiab] OR Jordanian[tiab] OR Iraq[tiab] OR Iraqi[tiab] OR Lebanon[tiab] OR lebanese[tiab] OR Saudi Arabia[tiab] OR Saudi Arabian[tiab] OR kuwait[tiab] OR Kuwaiti[tiab] OR United Arab Emirates[tiab] OR UAE[tiab] OR U.A.E.[tiab] OR djibouti[tiab] OR djiboutian[tiab] OR Syria[tiab] OR Syrian[tiab] OR muritania[tiab] OR muritanian[tiab] OR Yemen[tiab] OR Yemeni[tiab] OR Palestine[tiab] OR Palestinian[tiab]) |
| --- |

**Supplementary Table 1. Search Results: included articles, corresponding authors, their institutions and publishing journals**

| **Title** | **Corresponding Author** | **Institute** | **Journal** |
| --- | --- | --- | --- |
| Knowledge, attitude and beliefs towards HIV/AIDS among students of health institutes in Sana'a city | Al-Rabeei NA^58^ | University of Sanaa | EMHJ |
| AIDS awareness and attitudes among Yemeni young people living in high-risk areas | Al Serouri AW^59^ | University of Sanaa | EMHJ |
| Knowledge, attitudes and beliefs about HIV/AIDS in Sana'a, Yemen | Al Serouri AW^60^ | University of Sanaa | EMHJ |
| Physicians' knowledge, attitude and practice towards erectile dysfunction in Saudi Arabia | Abdulmohsen MF^61^ | King Faisal University | EMHJ |
| Knowledge, attitude and practices of Egyptian industrial and tourist workers towards HIV/AIDS | Kabbash IA^62^ | University of Tanta | EMHJ |
| Men's perceptions of and participation in family planning in Aqaba and Ma'an governorates, Jordan | Hamdan-Mansour A^63^ | University of Jordan | EMHJ |
| Knowledge, attitudes and practices of secondary-school pupils in Oman: II. reproductive health | Afifi M^64^ | League of Arab States | EMHJ |
| Evaluation of HIV voluntary counselling and testing services in Egypt. Part 1: client satisfaction | Kabbash IA^65^ | Tanta University | EMHJ |
| Evaluation of HIV voluntary counselling and testing services in Egypt. Part 2: service providers' satisfaction | Kabbash IA^66^ | Tanta University | EMHJ |
| Knowledge, attitudes, beliefs and practices in Lebanon concerning HIV/AIDS, 1996-2004 | Jurjus ARJG^67^ | Lebanese Health Society | EMHJ |
| HIV/AIDS knowledge and attitudes among alcohol and drug abusers in Egypt | Salama I^68^ | National Research Center | JEPHA |
| HIV/AIDS perceptions and risky behaviors in squatter areas in Cairo, Egypt | Kofi M^69^ | Suez canal University | JEPHA |
| University Students' knowledge, attitudes, and practices towards the National Premarital Screening Program of Saudi Arabia | Shilbayeh SAR^70^ | Princess Nourah Bint AbdulRahman University | JEPHA |
| Health-related quality of life in healthy children and adolescents of HIV-infected parents in Alexandria, Egypt | Ashry MH^71^ | Alexandria University | JEPHA |
| Knowledge and attitudes towards premarital counselling and examination | Dorgham LS^72^ | Menofia University | JEPHA |
| Multidisciplinary care model for HIV improves treatment outcome: a single-centre experience from the Middle East | Elgalib A^73^ | Directorate General for Disease Surveillance and Control | AIDS care |
| HIV stigma intervention in a low-HIV prevalence setting: a pilot study in an Egyptian healthcare facility | Lohiniva AL^74^ | Global Disease Detection and Response Program | AIDS Care |
| Continuing medical education needs regarding AIDS among Egyptian physicians in Alexandria, Egypt and in the Asir Region, Saudi Arabia | Sallam SA^75^ | Alexandria University | AIDS Care |
| Evaluation of a school-based HIV prevention intervention among Yemeni adolescents | Al Ryani B^76^ | Maastricht University | BMC Public health |
| HIV and AIDS related knowledge, sources of information, and reported need for further education among dental students in Sudan--a cross sectional study | Nasir EF^77^ | University of Science and Technology | BMC Public Health |
| HIV/AIDS-related attitudes and oral impacts on daily performances: a cross-sectional study of Sudanese adult dental patients | Nasir EF^78^ | University of Science and Technology | BMC Public Health |
| Knowledge and attitudes of undergraduate medical and non-medical students in Sultan Qaboos University toward Acquired Immune Deficiency Syndrome | Al-Jabri AA^79^ | Sultan Qaboos University | Saudi Med J |
| Exploring public knowledge and attitudes towards HIV/AIDS in Saudi Arabia. A survey of primary health care users | Al Ghanim SA^80^ | King Saud University | Saudi Med J |
| Knowledge and attitudes of paramedical students in Saudi Arabia toward HIV/AIDS | AlMazrou YY^81^ | MoH, KSA | Saudi Med J |
| HIV knowledge and stigmatization among adolescents in Yemeni schools | Al Ryani B^82^ | Maastricht University | Int Q Community Health Educ |
| Process evaluation of a three-year community-based peer education intervention for HIV prevention among Yemeni young people | Al Ryani B^83^ | Maastricht University | Int Q Community Health Educ |
| Insight into Jordanian thinking about HIV: knowledge of Jordanian men and women about HIV prevention | E Alkhasawneh^84^ | Sultan Qaboos University | J Assoc Nurses AIDS Care |
| HIV Stigma Toward People Living with HIV and Health Providers Associated with Their Care: Qualitative Interviews With Community Members in Egypt | Lohiniva AL^85^ | Global Disease Detection and Response Program | J Assoc Nurses AIDS Care |
| HIV/AIDS: dental assistants' self-reported knowledge and attitudes in Kuwait | AlMuzaini AAY^86^ | Kuwait University | Int Dent J |
| Men's knowledge, attitude, and barriers towards emergency contraception: A facility based cross-sectional study at King Saud University Medical City | Karim SI^87^ | King Saud University | Int Dent J |
| Missed opportunities for HIV testing in patients newly diagnosed with HIV in Morocco | Weiss L^88^ | Hôpital Européen Georges Pompidou | BMC Infect Dis |
| Scaling up combined community-based HIV prevention interventions targeting truck drivers in Morocco: effectiveness on HIV testing and counseling | Carrieri P^89^ | Aix Marseille University | BMC Infect Dis |
| Break the silence: HIV/AIDS knowledge, attitudes, and educational needs among Arab university students in United Arab Emirates | Barss P^90^ | United Arab Emirates University | J Adolesc Health |
| Learning about Barriers to Care for People Living with HIV in Egypt: A Qualitative Exploratory Study | Lohiniva AL^91^ | Global Disease Detection & Response Program | JIAPAC |
| Should an inquiry about sexual health, as a reflection of vascular health, be part of routine physicals for young men? Results from an outpatient study | Al Turki YA^92^ | King Saud University | Int J Impot Res |
| The impact of a lecture on AIDS on knowledge, attitudes and beliefs of male school-age adolescents in the Asir Region of southwestern Saudi Arabia | Abolfotouh MA^93^ | King Saud University | J Community Health |
| A Survey on Sexual Counseling for Patients with Cardiac Disease Among Nurses in Jordan | Abed MA^94^ | Hashemite University | J Cardiovasc Nurs |
| An assessment of HIV patient's adherence to treatment and need for pharmaceutical care in Jordan | Mukattash TL^95^ | Jordan University of Science & Technology | In. J Clin. Pract |
| Assessing HIV/AIDS Knowledge, Awareness, and Attitudes among Senior High School Students in Kuwait | AlHasawi A^96^ | Infectious Diseases Hospital, MoH | Med Princ Pract |
| Violence, abuse, alcohol and drug use, and sexual behaviors in street children of Greater Cairo and Alexandria, Egypt | Nada KH^97^ | Population Council, West Asia and North Africa Regional Office | AIDS |
| Process evaluation of school-based peer education for HIV prevention among Yemeni adolescents | Al Ryani B^98^ | Maastricht University | Journal of Social Aspects of HIV/AIDS |
| Knowledge, attitudes, beliefs, and practices about HIV/AIDS in Kuwait | Al Owaish R^99^ | University of Kuwait | AIDS Educ Prev |
| The Awareness of the Human Papillomavirus Infection and Oropharyngeal Cancer in People to Improve the Health Care System at Al Qunfudhah Region, Kingdom of Saudi Arabia | Alshehri AM^100^ | King Khalid University | J Healthc Eng |
| Knowledge and attitudes toward HIV/AIDS among the general population of Jeddah, Saudi Arabia | Alwafi HA^101^ | King Abdulaziz University | J. Infect. Public Health |
| Attitudes and practices of oncologists toward fertility preservation | Arafa MA^102^ | King Khalid University | J Pediatr Hematol Oncol |
| Arab dentists' refusal to treat HIV positive patients: a survey of recently graduated dentists from three Arab dental schools | El Tantawi M^103^ | University of Damam | Acta Odontol. Scand |
| An Exploratory Study of HIV Risk Behaviors and Testing among Male Sex Workers in Beirut, Lebanon | Aunon F^104^ | RAND corporation, Santa Monica | Soc Work Public Health |
| Stigmatization of persons with HIV/AIDS in Saudi Arabia | Badahdah AM^105^ | University of North Dakota | J Transcult Nurs |
| Knowledge and Perception Regarding the Development and Acceptability of Male Contraceptives Among Pharmacists: A Mixed Sequential Method | Barakat M^106^ | Applied Science Private University | AJMH |
| The realities of HIV prevention. A closer look at facilitators and challenges faced by HIV prevention programmes in Sudan and Yemen | Bashir F^107^ | Umeå University | Global Health Action |
| The association between medical spending and health status: A study of selected African countries | Bein M^108^ | Cyprus International University | Malawi Med J |
| HIV/AIDS related knowledge among school-going adolescents from the Middle East and North Africa | Boneberger A^109^ | University Hospital of Munich | Sexual Health |
| Family physicians' attitude and practice of infertility management at primary care--Suez Canal University, Egypt | Nour-Eldein H^110^ | Suez Canal University | Pan Afr Med J |
| The cascade of HIV care in Oman, 2015-2018: A population-based study from the Middle East | Elgalib A^111^ | Directorate General for Disease Surveillance and Control | Int J Infect Dis |
| Knowledge and attitudes towards HIV/AIDS amongst Kuwait University dental students | Sundaram DB^112^ | Kuwait University | Eur J Dent Educ |
| Reaching Egyptian Gays Using Social Media: A Comprehensive Health Study and a Framework for Future Research | Elmahy AG^113^ | Alexandria University | J Homosex |
| The cost to health services of human immunodeficiency virus (HIV) co-infection among tuberculosis patients in Sudan | El-Sony AI^114^ | National Tuberculosis Program | Health policy |
| A qualitative study of Egyptian school nurses' attitudes and experiences toward sex and relationship education | Hayter M^115^ | King Abdullah Medical City | J Sch Nurs |
| Human papillomavirus knowledge and vaccine acceptability among male medical students in Saudi Arabia | Faris NJ^116^ | King Abdulaziz University | Hum Vaccines Immunother |
| Knowledge of Human Papillomavirus (HPV) and Oropharyngeal Cancer and Acceptability of the HPV Vaccine among Dental Students | Faris NJ^117^ | King Abdulaziz University | APJCP |
| Survey of HIV/AIDS knowledge and attitudes of Kuwaiti family physicians | Fido A^118^ | Kuwait University | Fam Pract |
| Health care professionals' attitudes towards youth-friendly sexual and reproductive health services in Jordan: a cross-sectional study of physicians, midwives and nurses | Gausman J^119^ | Harvard TH Chan School of Public Health | Reprod Health |
| A study on the knowledge and practice of contraception among men in the United Arab Emirates | Ghazal-Aswad S^120^ | United Arab Emirates University | J Fam Plann Reprod Health Care |
| Knowledge, attitudes and awareness regarding fertility preservation among oncologists and clinical practitioners in Lebanon | Abiad GG^121^ | American University of Beirut Medical Center | Hum Fertil |
| Assessing Knowledge of, and Attitudes to, HIV/AIDS among University Students in the United Arab Emirates | Haroun D^122^ | UNICEF Gulf Area Office | PLOS one |
| Knowledge and attitudes of Jordanian nurses towards patients with HIV/AIDS: findings from a nationwide survey | Hassan ZM^123^ | Hashemite University | Issues Ment Health Nurs |
| Planning STI/HIV prevention among refugees and mobile populations: situation assessment of Sudanese refugees | Holt BY^124^ | University of California | Disasters |
| Globalization and reproductive tourism in the United Arab Emirates | Inhorn MC^125^ | Yale University | APJPH |
| Knowledge, attitudes and practice of condom use among males aged (15-49) years in Erbil Governorate | Zangana JM^126^ | Hawler Medical University | Glob J Health Sci |
| HIV/AIDS-related stigma and discrimination among health care workers at Tanta University Hospitals, Egypt | Abo Ali EA^127^ | Tanta University | Environ. Sci. Pollut. Res. |
| Healthcare practitioners’ experiences in delivering sexual and reproductive health services to unmarried adolescent clients in Jordan: results from a cross-sectional survey | Gausman J^128^ | Harvard Chan School of Public Health | BMC Health Serv Res |
| Youth-friendly reproductive health services in Jordan from the perspective of the youth: a descriptive qualitative study | Khalaf IA^129^ | University of Jordan | Scand J Caring Sci |
| HIV/AIDS in Syria and the response of the National AIDS Program during the war | Ghaddar A^130^ | Lebanese International University | Sex Transm Infect |
| Civil war and male infertility in Lebanon | Abu-Musa AA^131^ | American University of Beirut | Fertil Steril |
| Access to HIV Care and Resilience in a Long-Term Conflict Setting: A Qualitative Assessment of the Experiences of Living with Diagnosed HIV in Mogadishu, Somali | Kulane A^132^ | Karolinska Institute | IJERPH |
| The assessment of seafarers' knowledge, attitudes and practices related to STI/HIV/AIDS in northern Morocco | Laraqui CH^133^ | Ecole Supérieure D’ingénierie De La Santé | IMH |
| Community-led HIV self-testing for men who have sex with men in Lebanon: lessons learned and impact of COVID-19 | Maatouk I^134^ | Clemenceau Medical Center | bmc health research policy and systems |
| Knowledge and attitudes towards AIDS among primary health care physicians in the Asir Region, Saudi Arabia | Mahfouz AA^135^ | King Saud University | R Soc Health J |
| Knowledge and Attitudes of Doctors Toward People Living With HIV/AIDS in Saudi Arabia | Memish ZA^136^ | Ministry of Health, KSA | JAIDS |
| Male attitudes towards family planning in Khartoum, Sudan | Mumford SD^137^ | Research Triangle Park | J Biosoc Sci |
| Examining the Attitudes and Behaviors of Health-care Providers Toward LGBT Patients in Lebanon | Naal H^138^ | Haigazian University | J Homosex |
| Knowledge about HIV/AIDS among high school students in Erbil city/Iraq | Othman SM^139^ | Hawler Medical University | Glob J Health Sci |
| Knowledge of and attitudes towards HIV/AIDS: a survey among dental students in Ajman, UAE | Premadasa G^140^ | Gulf Medical University | J Investig Clin Dent |
| Oncologists' knowledge and practice towards sperm cryopreservation in Arabic communities | Rabah DM^141^ | King Saud University | J Cancer Surviv |
| Attitudes Towards and Practice Of Sexuality Among University Students In Lebanon | Salameh P^142^ | Lebanese University | Journal of Biosocial Science |
| Impact of health education program on knowledge about AIDS and HIV transmission in students of secondary schools in Buraidah city, Saudi Arabia: an exploratory study | Saleh MA^143^ | King Fahad Specialist Hospital | J Fam Community Med |
| Reproductive health and HIV awareness among newly married Egyptian couples without formal education | Saleh WF^144^ | Cairo University | Int J Gynaecol Obstet |
| Knowledge, Attitudes and Practices Related to HIV Stigma and Discrimination Among Healthcare Workers in Oman | Al-Abri SS^145^ | Ministry of Health | Sultan Qaboos Univ Med J |
| Jordan's 2002 to 2012 Fertility Stall and Parallel USAID Investments in Family Planning: Lessons From an Assessment to Guide Future Programming | Spindler E^146^ | Georgetown University | Glob. health, Sci. Pract. |
| An evaluation of the population and development program in Egypt | Stycos JM^147^ | Cornell University | Demography |
| Filling the Knowledge Gap: Measuring HIV Prevalence and Risk Factors among Men Who Have Sex with Men and Female Sex Workers in Tripoli, Libya | Berendes S^148^ | London school of hygeine and tropical medicine | PLOS |
| Fertility and family planning in Jordan: results from the 1985 Jordan Husbands' Fertility Survey | Warren CW^149^ | Centers for Chronic Disease Control | Stud Fam Plann |
| Cross-cultural comparison of fertility specific quality of life in German, Hungarian and Jordanian couples attending a fertility center | Wischmann T^150^ | Ruprecht-Karls University | Health Qual. |
| Sexual information needs of Arab-Muslim patients with cardiac problems | Akhu-Zaheya LM^151^ | Jordan University of Science & Technology | Eur J Cardiovasc Nurs |
| Perceived Knowledge, Practices, Attitudes and Beliefs of Jordanian Nurses Toward Sexual Health Assessment of Patients with Coronary Artery Diseases | Maribbay GL^152^ | Al-Ghad International Colleges for Applied Medical Sciences | Sex Disabil |
| Knowledge, attitudes, and perception patterns of contraception methods: Cross-sectional study among Saudi males | Binsaleh S^153^ | King Saud University | Urology Annals |
| What are the needs of infertile Moroccan couples in Assisted Reproductive Technology? Exploratory qualitative study in the first fertility public center in Morocco | Zaidouni A^154^ | University Mohamed V | Bangladesh J. Medical Sci |
| Resource Utilization and Caring Cost of People Living with Human Immunodeficiency Virus (PLHIV) in Saudi Arabia: A Tertiary Care University Hospital Experience | Al-Omar HA^155^ | King Saud University | Healthcare |

MoH: Ministry of Health; KSA; Acta Odontol. Scand: Acta Odontologica Scandinavica; AJMH: American Journal of Men’s Health; APJCP: Asian pacific journal of cancer prevention; APJPH: Asian pacific journal of public health; Bangladesh J Medical Sci: Bangladesh Journal of Medical Science; BMC Health Serv Res: BMC Health Services Research; BMC Infect Dis: BMC Infectious Diseases; EMHJ: Eastern Mediterranean Health Journal; Environ. Sci Pollut Res Environmental Science and Pollution Research; Eur J Cardiovasc Nurs: European journal of cardiovascular nursing; Eur J Dent Educ: European Journal of Dental Education; Fam Pract: Family practice; Fertil Steril: Fertility & Sterility; Glob health Sci Pract: Global Health: Science and Practice; Glob J Health Sci: Global Journal of Health Science; Health Qual: Health and Quality of Life Outcome; Hum Fertil: human fertility; Issues Ment Health Nurs: Issues in Mental Health Nursing; Hum Vaccines Immunother: Human Vaccines & Immunotherapeutics; IJERPH: International Journal of Environmental Research and Public Health; IMH**:** International maritime health; Int Dent J: International Dental Journal; Int Dent J: International Dental Journal; AIDS Educ Prev: AIDS Education & prevention; Int J Clin Pract; The international journal of clinical practice; Int J Gynaecol Obstet: International journal of gynaecology and obstetrics; Int J Impot Res: International Journal of Impotence Research; Int J Infect Dis: International Journal of Infectious Diseases; Int Q Community Health Educ: nternational Quarterly of Community Health Education; J Adolesc Reprod Health: reproductive health; J Assoc Nurses AIDS Care: Journal of the Association of nurses in AIDS Care; J Biosoc Sci: Journal of Biosocial Science; J Cancer Surviv: Journal of Cancer Survivorship; J Cardiovasc Nurs: Journal of Cardiovascular Nursing; J Community Health: Journal of Community Health; J Fam Community Med: Journal of family and community medicine; J Healthc Eng: Journal of Healthcare Engineering; J Homosex: Journal of Homosexuality; J Infect. Public Health: Journal of infection and public health; J Investig Clin Dent: Journal of Investigative and Clinical Dentistry; J Pediatr Hematol Oncol: Journal of pediatric hematology and oncology; J Sch Nurs: Journal of School Nursing; J Transcult Nurs: Journal of Transcultural Nursing; JAIDS: Journal of acquired immune deficiency syndromes; JEPHA: Journal of the Egyptian Public Health Association; JIAPAC: Journal of International Association of Providers of AIDS Care; Malawi Med J: Malawi Medical Journal; Med Princ Pract: Medical principles and practice; Pan Afr Med J: Pan African Medical Journal; R Soc Health J: Journal of the Royal Society of Health; Saudi Med J: Saudi Medical Journal; Scand J Caring Sci: scandinavian journal of caring sciences; Sex Disabil: Sexuality & Disability; Sex. Transm Infect: Sexually Transmitted Infections; Soc Work Public Health: Social work on public health; Stud Fam Plann: Studies in Family Planning; Sultan Qaboos Univ Med J: Sultan Qaboos University Medical Journal; The Journal of Family Planning and Reproductive Health Care

**References**

1. Al-Rabeei NA, Dallak AM, Al-Awadi FG. Knowledge, attitude and beliefs towards HIV/AIDS among students of health institutes in Sana'a city. East Mediterr Health J. 2012;18(3):221-6. doi: 10.26719/2012.18.3.221. PMID: 22574474.
2. Al-Serouri AW, Anaam M, Al-Iryani B, Al Deram A, Ramaroson S. AIDS awareness and attitudes among Yemeni young people living in high-risk areas. East Mediterr Health J. 2010;16(3):242-50. PMID: 20795436.
3. Al-Serouri AW, Takioldin M, Oshish H, Aldobaibi A, Abdelmajed A. Knowledge, attitudes and beliefs about HIV/AIDS in Sana'a, Yemen. East Mediterr Health J. 2002;8(6):706-15. PMID: 15568447.
4. Abdulmohsen MF, Abdulrahman IS, Al-Khadra AH, Bahnassy AA, Taha SA, Kamal BA, Al-Rubaish AM, Ai-Elq AH. Physicians' knowledge, attitude and practice towards erectile dysfunction in Saudi Arabia. East Mediterr Health J. 2004;10(4-5):648-54. PMID: 16335658.
5. El-Sayyed N, Kabbash IA, El-Gueniedy M. Knowledge, attitude and practices of Egyptian industrial and tourist workers towards HIV/AIDS. East Mediterr Health J. 2008;14(5):1126-35. PMID: 19161085.
6. Hamdan-Mansour AM, Malkawi AO, Sato T, Hamaideh SH, Hanouneh SI. Men's perceptions of and participation in family planning in Aqaba and Ma'an governorates, Jordan. East Mediterr Health J. 2016;22(2):124-32. doi: 10.26719/2016.22.2.124. PMID: 27180740.
7. Jaffer YA, Afifi M, Al Ajmi F, Alouhaishi K. Knowledge, attitudes and practices of secondary-school pupils in Oman: II. reproductive health. East Mediterr Health J. 2006;12(1-2):50-60. PMID: 17037221.
8. Kabbash IA, Hassan NM, Al-Nawawy AN, Attalla AA, Mekheimer SI. Evaluation of HIV voluntary counselling and testing services in Egypt. Part 1: client satisfaction. East Mediterr Health J. 2010;16(5):481-90. PMID: 20799546.
9. Kabbash IA, Mekheimer Sl, Hassan NM, Al-Nawawy AN, Attalla AA. Evaluation of HIV voluntary counselling and testing services in Egypt. Part 2: service providers' satisfaction. East Mediterr Health J. 2010;16(5):491-7. PMID: 20799547.
10. Kahhaleh JG, El Nakib M, Jurjus AR. Knowledge, attitudes, beliefs and practices in Lebanon concerning HIV/AIDS, 1996-2004. East Mediterr Health J. 2009;15(4):920-33. PMID: 20187544.
11. Salama II, Kotb NK, Hemeda SA, Zaki F. HIV/AIDS knowledge and attitudes among alcohol and drug abusers in Egypt. J Egypt Public Health Assoc. 1998;73(5-6):479-500. PMID: 17217020.
12. Shama M, Fiala LE, Abbas MA. HIV/AIDS perceptions and risky behaviors in squatter areas in Cairo, Egypt. J Egypt Public Health Assoc. 2002;77(1-2):173-200. PMID: 17219897.
13. Melaibari M, Shilbayeh S, Kabli A. University Students' knowledge, attitudes, and practices towards the National Premarital Screening Program of Saudi Arabia. J Egypt Public Health Assoc. 2017;92(1):36-43. doi: 10.21608/epx.2017.7008. PMID: 29924926.
14. Ashry M, Ziady H, Hameed M, Mohammed F. Health-related quality of life in healthy children and adolescents of HIV-infected parents in Alexandria, Egypt. J Egypt Public Health Assoc. 2017;92(4):212-219. doi: 10.21608/EPX.2018.22042. PMID: 30612381.
15. Eshra DK, Dorgham LS, el-Sherbini AF. Knowledge and attitudes towards premarital counselling and examination. J Egypt Public Health Assoc. 1989;64(1-2):1-15. PMID: 2520141.
16. Elgalib A, Al-Sawafi H, Kamble B, Al-Harthy S, Al-Sariri Q. Multidisciplinary care model for HIV improves treatment outcome: a single-centre experience from the Middle East. AIDS Care. 2018;30(9):1114-1119. doi: 10.1080/09540121.2018.1479028. Epub 2018 May 24. PMID: 29792340.
17. Lohiniva AL, Benkirane M, Numair T, Mahdy A, Saleh H, Zahran A, Okasha O, Talaat M, Kamal W. HIV stigma intervention in a low-HIV prevalence setting: a pilot study in an Egyptian healthcare facility. AIDS Care. 2016;28(5):644-52. doi: 10.1080/09540121.2015.1124974.
18. Sallam SA, Mahfouz AA, Alakija W, al-Erian RA. Continuing medical education needs regarding AIDS among Egyptian physicians in Alexandria, Egypt and in the Asir Region, Saudi Arabia. AIDS Care. 1995;7(1):49-54. doi: 10.1080/09540129550126957. PMID: 7748910.
19. Al-Iryani B, Basaleem H, Al-Sakkaf K, Crutzen R, Kok G, van den Borne B. Evaluation of a school-based HIV prevention intervention among Yemeni adolescents. BMC Public Health. 2011;11:279. doi: 10.1186/1471-2458-11-279. PMID: 21548968; PMCID: PMC3112119.
20. Nasir EF, Astrøm AN, David J, Ali RW. HIV and AIDS related knowledge, sources of information, and reported need for further education among dental students in Sudan--a cross sectional study. BMC Public Health. 2008;8:286. doi: 10.1186/1471-2458-8-286. PMID: 18702806; PMCID: PMC2527565.
21. Nasir EF, Marthinussen MC, Åstrøm AN. HIV/AIDS-related attitudes and oral impacts on daily performances: a cross-sectional study of Sudanese adult dental patients. BMC Health Serv Res. 2013;13:335. doi: 10.1186/1472-6963-13-335. PMID: 23971974; PMCID: PMC3765642.
22. Al-Jabri AA, Al-Abri JH. Knowledge and attitudes of undergraduate medical and non-medical students in Sultan Qaboos University toward acquired immune deficiency syndrome. Saudi Med J. 2003;24(3):273-7. PMID: 12704503.
23. Al-Ghanim SA. Exploring public knowledge and attitudes towards HIV/AIDS in Saudi Arabia. A survey of primary health care users. Saudi Med J. 2005;26(5):812-8. PMID: 15951875.
24. Al-Mazrou YY, Abouzeid MS, Al-Jeffri MH. Knowledge and attitudes of paramedical students in Saudi Arabia toward HIV/AIDS. Saudi Med J. 2005;26(8):1183-9. PMID: 16127510.
25. Al-Iryani B, Raja'a YA, Kok G, Van Den Borne B. HIV knowledge and stigmatization among adolescents in Yemeni schools. Int Q Community Health Educ. 2009-2010;30(4):311-20. doi: 10.2190/IQ.30.4.c. PMID: 21273165.
26. Al-Iryani B, Al-Sakkaf K, Basaleem H, Kok G, van den Borne B. Process evaluation of a three-year community-based peer education intervention for HIV prevention among Yemeni young people. Int Q Community Health Educ. 2010;31(2):133-54. doi: 10.2190/IQ.31.2.c. PMID: 21840812.
27. Alkhasawneh E, McFarland W, Mandel J, Seshan V. Insight into Jordanian thinking about HIV: knowledge of Jordanian men and women about HIV prevention. J Assoc Nurses AIDS Care. 2014;25(1):e1-9. doi: 10.1016/j.jana.2013.06.001. Epub 2013 Oct 14. PMID: 24135312.
28. Lohiniva AL, Kamal W, Benkirane M, Numair T, Abdelrahman M, Saleh H, Zahran A, Talaat M, Kandeel A. HIV Stigma Toward People Living With HIV and Health Providers Associated With Their Care: Qualitative Interviews With Community Members in Egypt. J Assoc Nurses AIDS Care. 2016;27(2):188-98. doi: 10.1016/j.jana.2015.11.007. Epub 2015 Dec 2. PMID: 26718817.
29. AlMuzaini AA, Yahya AS, Ellepola AN, Sharma PN. HIV/AIDS: dental assistants' self-reported knowledge and attitudes in Kuwait. Int Dent J. 2015;65(2):96-102. doi: 10.1111/idj.12136. Epub 2014 Oct 24. PMID: 25345503.
30. Karim SI, Irfan F, Saad H, Alqhtani M, Alsharhan A, Alzhrani A, Alhawas F, Alatawi S, Alassiri M, M A Ahmed A. Men's knowledge, attitude, and barriers towards emergency contraception: A facility based cross-sectional study at King Saud University Medical City. PLoS One. 2021;16(4):e0249292. doi: 10.1371/journal.pone.0249292. PMID: 33901184; PMCID: PMC8075244.
31. Marih L, Sawras V, Pavie J, Sodqi M, Malmoussi M, Tassi N, Bensghir R, Nani S, Lahsen AO, Laureillard D, El Filali KM, Champenois K, Weiss L. Missed opportunities for HIV testing in patients newly diagnosed with HIV in Morocco. BMC Infect Dis. 2021;21(1):48. doi: 10.1186/s12879-020-05711-2. PMID: 33430783; PMCID: PMC7802172.
32. Himmich H, Ouarsas L, Hajouji FZ, Lions C, Roux P, Carrieri P. Scaling up combined community-based HIV prevention interventions targeting truck drivers in Morocco: effectiveness on HIV testing and counseling. BMC Infect Dis. 2015;15:208. doi: 10.1186/s12879-015-0936-6. PMID: 25940535; PMCID: PMC4425859.
33. Gańczak M, Barss P, Alfaresi F, Almazrouei S, Muraddad A, Al-Maskari F. Break the silence: HIV/AIDS knowledge, attitudes, and educational needs among Arab university students in United Arab Emirates. J Adolesc Health. 2007;40(6):572.e1-8. doi: 10.1016/j.jadohealth.2007.01.011. Epub 2007 Mar 26. PMID: 17531765
34. Abdelrahman I, Lohiniva AL, Kandeel A, Benkirane M, Atta H, Saleh H, El Sayed N, Talaat M. Learning about Barriers to Care for People Living with HIV in Egypt: A Qualitative Exploratory Study. J Int Assoc Provid AIDS Care. 2015;14(2):141-7. doi: 10.1177/2325957413488180. Epub 2013 Jun 21. PMID: 23792709.
35. Abdulah Al Turki Y. Should an inquiry about sexual health, as a reflection of vascular health, be part of routine physicals for young men? Results from an outpatient study. Int J Impot Res. 2009;21(6):362-5. doi: 10.1038/ijir.2009.36. Epub 2009 Aug 20. PMID: 19693020
36. Abolfotouh MA. The impact of a lecture on AIDS on knowledge, attitudes and beliefs of male school-age adolescents in the Asir Region of southwestern Saudi Arabia. J Community Health. 1995;20(3):271-81. doi: 10.1007/BF02260410. PMID: 7657860.
37. Abu Ali RM, Abed MA, Khalil AA, Al-Kloub MI, Ashour AF, Alnsour IA. A Survey on Sexual Counseling for Patients With Cardiac Disease Among Nurses in Jordan. J Cardiovasc Nurs. 2018;33(5):467-473. doi: 10.1097/JCN.0000000000000472. PMID: 29601371
38. Al Tall YR, Mukattash TL, Sheikha H, Jarab AS, Nusair MB, Abu-Farha RK. An assessment of HIV patient's adherence to treatment and need for pharmaceutical care in Jordan. Int J Clin Pract. 2020;74(7):e13509. doi: 10.1111/ijcp.13509. Epub 2020 Apr 29. PMID: 32279382.
39. Alhasawi A, Grover SB, Sadek A, Ashoor I, Alkhabbaz I, Almasri S. Assessing HIV/AIDS Knowledge, Awareness, and Attitudes among Senior High School Students in Kuwait. Med Princ Pract. 2019;28(5):470-476. doi: 10.1159/000500307. Epub 2019 Apr 16. PMID: 30995643; PMCID: PMC6771065.
40. Nada KH, Suliman el DA. Violence, abuse, alcohol and drug use, and sexual behaviors in street children of Greater Cairo and Alexandria, Egypt. AIDS. 2010;24 Suppl 2:S39-44. doi: 10.1097/01.aids.0000386732.02425.d1. PMID: 20610947.
41. Al-Iryani B, Basaleem H, Al-Sakkaf K, Kok G, van den Borne B. Process evaluation of school-based peer education for HIV prevention among Yemeni adolescents. SAHARA J. 2013;10(1):55-64. doi: 10.1080/17290376.2012.745294. Epub 2013 Jun 18. PMID: 23777570; PMCID: PMC3914420.
42. Al-Owaish R, Moussa MA, Anwar S, al-Shoumer H, Sharma P. Knowledge, attitudes, beliefs, and practices about HIV/AIDS in Kuwait. AIDS Educ Prev. 1999;11(2):163-73. PMID: 10214499.
43. Alshehri AM et al. The Awareness of the Human Papillomavirus Infection and Oropharyngeal Cancer in People to Improve the Health Care System at Al Qunfudhah Region, Kingdom of Saudi Arabia. J Healthc Eng. 2021;2021:5185075. doi: 10.1155/2021/5185075. PMID: 34712459; PMCID: PMC8548161.
44. Alwafi HA, Meer AMT, Shabkah A, Mehdawi FS, El-Haddad H, Bahabri N, Almoallim H. Knowledge and attitudes toward HIV/AIDS among the general population of Jeddah, Saudi Arabia. J Infect Public Health. 2018;11(1):80-84. doi: 10.1016/j.jiph.2017.04.005.
45. Arafa MA, Rabah DM. Attitudes and practices of oncologists toward fertility preservation. J Pediatr Hematol Oncol. 2011;33(3):203-7. doi: 10.1097/MPH.0b013e3182068047. PMID: 21336166.
46. Arheiam A, El Tantawi M, Al-Ansari A, Ingafou M, El Howati A, Gaballah K, AbdelAziz W. Arab dentists' refusal to treat HIV positive patients: a survey of recently graduated dentists from three Arab dental schools. Acta Odontol Scand. 2017;75(5):355-360. doi: 10.1080/00016357.2017.1316867.
47. Aunon FM, Wagner GJ, Maher R, Khouri D, Kaplan RL, Mokhbat J. An Exploratory Study of HIV Risk Behaviors and Testing among Male Sex Workers in Beirut, Lebanon. Soc Work Public Health. 2015;30(4):373-84. doi: 10.1080/19371918.2014.979274. PMID: 25950906; PMCID: PMC4562212.
48. Badahdah AM. Stigmatization of persons with HIV/AIDS in Saudi Arabia. J Transcult Nurs. 2010;21(4):386-92. doi: 10.1177/1043659609360873. Epub 2010 Jun 30. PMID: 20592063.
49. Barakat M, Thiab S, Thiab S, Al-Qudah RA, Akour A. Knowledge and Perception Regarding the Development and Acceptability of Male Contraceptives Among Pharmacists: A Mixed Sequential Method. Am J Mens Health. 2022;16(1):15579883221074855. doi: 10.1177/15579883221074855. PMID: 35135388; PMCID: PMC8832602.
50. Bashir F, Ba Wazir M, Schumann B, Lindvall K. The realities of HIV prevention. A closer look at facilitators and challenges faced by HIV prevention programmes in Sudan and Yemen. Glob Health Action. 2019;12(1):1659098. doi: 10.1080/16549716.2019.1659098. PMID: 31496422; PMCID: PMC6746302.
51. Bein M, Coker-Farrell EY. The association between medical spending and health status: A study of selected African countries. Malawi Med J. 2020;32(1):37-44. doi: 10.4314/mmj.v32i1.8. PMID: 32733658; PMCID: PMC7366161.
52. Boneberger A, Rückinger S, Guthold R, Kann L, Riley L. HIV/AIDS related knowledge among school-going adolescents from the Middle East and North Africa. Sex Health. 2012;9(2):196-8. doi: 10.1071/SH11054. PMID: 22498168.
53. Eldein HN. Family physicians' attitude and practice of infertility management at primary care--Suez Canal University, Egypt. Pan Afr Med J. 2013;15:106. doi: 10.11604/pamj.2013.15.106.1762. PMID: 24244792; PMCID: PMC3828067.
54. Elgalib A, Shah S, Al-Habsi Z, Al-Fouri M, Lau R, Al-Kindi H, Al-Rawahi B, Al-Abri S. The cascade of HIV care in Oman, 2015-2018: A population-based study from the Middle East. Int J Infect Dis. 2020;90:28-34. doi: 10.1016/j.ijid.2019.09.017. Epub 2019 Sep 27. PMID: 31568859.
55. Ellepola AN, Joseph BK, Sundaram DB, Sharma PN. Knowledge and attitudes towards HIV/AIDS amongst Kuwait University dental students. Eur J Dent Educ. 2011;15(3):165-71. doi: 10.1111/j.1600-0579.2010.00652.x. Epub 2011 Jan 31. PMID: 21762321.
56. Elmahy AG. Reaching Egyptian Gays Using Social Media: A Comprehensive Health Study and a Framework for Future Research. J Homosex. 2018;65(13):1867-1876. doi: 10.1080/00918369.2017.1395658.
57. El-Sony AI. The cost to health services of human immunodeficiency virus (HIV) co-infection among tuberculosis patients in Sudan. Health Policy. 2006;75(3):272-9. doi: 10.1016/j.healthpol.2005.01.006. Epub 2005 Dec 2. PMID: 16325960.
58. Farrag S, Hayter M. A qualitative study of Egyptian school nurses' attitudes and experiences toward sex and relationship education. J Sch Nurs. 2014;30(1):49-56. doi: 10.1177/1059840513506941. Epub 2013 Oct 8. PMID: 24106180.
59. Farsi NJ, Baharoon AH, Jiffri AE, Marzouki HZ, Merdad MA, Merdad LA. Human papillomavirus knowledge and vaccine acceptability among male medical students in Saudi Arabia. Hum Vaccin Immunother. 2021;17(7):1968-1974. doi: 10.1080/21645515.2020.1856597. Epub 2021 Jan 31. PMID: 33522406; PMCID: PMC8189128.
60. Farsi NJ, Al Sharif S, Al Qathmi M, Merdad M, Marzouki H, Merdad L. Knowledge of Human Papillomavirus (HPV) and Oropharyngeal Cancer and Acceptability of the HPV Vaccine among Dental Students. Asian Pac J Cancer Prev. 2020;21(12):3595-3603. doi: 10.31557/APJCP.2020.21.12.3595. PMID: 33369457; PMCID: PMC8046298.
61. Fido A, Al Kazemi R. Survey of HIV/AIDS knowledge and attitudes of Kuwaiti family physicians. Fam Pract. 2002;19(6):682-4. doi: 10.1093/fampra/19.6.682. PMID: 12429674.
62. Gausman J, Othman A, Al-Qotob R, Shaheen A, Abu Sabbah E, Aldiqs M, Hamad I, Dabobe M, Langer A. Health care professionals' attitudes towards youth-friendly sexual and reproductive health services in Jordan: a cross-sectional study of physicians, midwives and nurses. Reprod Health. 2021;18(1):84. doi: 10.1186/s12978-021-01137-4. PMID: 33882951; PMCID: PMC8059015.
63. Ghazal-Aswad S, Zaib-Un-Nisa S, Rizk DE, Badrinath P, Shaheen H, Osman N. A study on the knowledge and practice of contraception among men in the United Arab Emirates. J Fam Plann Reprod Health Care. 2002;28(4):196-200. doi: 10.1783/147118902101196559. PMID: 12419060.
64. Ghazeeri G, Zebian D, Nassar AH, Harajly S, Abdallah A, Hakimian S, Skaiff B, Abbas HA, Awwad J. Knowledge, attitudes and awareness regarding fertility preservation among oncologists and clinical practitioners in Lebanon. Hum Fertil (Camb). 2016;19(2):127-33. doi: 10.1080/14647273.2016.1193636.
65. Haroun D, El Saleh O, Wood L, Mechli R, Al Marzouqi N, Anouti S. Assessing Knowledge of, and Attitudes to, HIV/AIDS among University Students in the United Arab Emirates. PLoS One. 2016;11(2):e0149920. doi: 10.1371/journal.pone.0149920. PMID: 26913902; PMCID: PMC4767799.
66. Hassan ZM, Wahsheh MA. Knowledge and attitudes of Jordanian nurses towards patients with HIV/AIDS: findings from a nationwide survey. Issues Ment Health Nurs. 2011;32(12):774-84. doi: 10.3109/01612840.2011.610562. PMID: 22077750.
67. Holt BY, Effler P, Brady W, Friday J, Belay E, Parker K, Toole M. Planning STI/HIV prevention among refugees and mobile populations: situation assessment of Sudanese refugees. Disasters. 2003;27(1):1-15. doi: 10.1111/1467-7717.00216. PMID: 12703149
68. Inhorn MC, Shrivastav P. Globalization and reproductive tourism in the United Arab Emirates. Asia Pac J Public Health. 2010;22(3 Suppl):68S-74S. doi: 10.1177/1010539510373007. PMID: 20566536.
69. Ismael AS, Sabir Zangana JM. Knowledge, attitudes and practice of condom use among males aged (15-49) years in Erbil Governorate. Glob J Health Sci. 2012;4(4):27-36. doi: 10.5539/gjhs.v4n4p27. PMID: 22980338; PMCID: PMC4776938.
70. Kabbash IA, Abo Ali EA, Elgendy MM, Abdrabo MM, Salem HM, Gouda MR, Elbasiony YS, Elboshy N, Hamed M. HIV/AIDS-related stigma and discrimination among health care workers at Tanta University Hospitals, Egypt. Environ Sci Pollut Res Int. 2018;25(31):30755-30762. doi: 10.1007/s11356-016-7848-x.
71. Kapoor NR, Langer A, Othman A, Gausman J. Healthcare practitioners experiences in delivering sexual and reproductive health services to unmarried adolescent clients in Jordan: results from a cross-sectional survey. BMC Health Serv Res. 2022;22(1):31. doi: 10.1186/s12913-021-07415-y. PMID: 34986832; PMCID: PMC8734334.
72. Khalaf I, Abu Moghli F, Froelicher ES. Youth-friendly reproductive health services in Jordan from the perspective of the youth: a descriptive qualitative study. Scand J Caring Sci. 2010;24(2):321-31. doi: 10.1111/j.1471-6712.2009.00723.x. Epub 2010 Mar 10. PMID: 20233355.
73. Khamis J, Ghaddar A. HIV/AIDS in Syria and the response of the National AIDS Program during the war. Sex Transm Infect. 2018;94(3):173. doi: 10.1136/sextrans-2017-053438. PMID: 29764976.
74. Kobeissi L, Inhorn MC, Hannoun AB, Hammoud N, Awwad J, Abu-Musa AA. Civil war and male infertility in Lebanon. Fertil Steril. 2008;90(2):340-5. doi: 10.1016/j.fertnstert.2007.06.061. Epub 2007 Oct 24. PMID: 17919611.
75. Kulane A, Owuor JOA, Sematimba D, Abdulahi SA, Yusuf HM, Mohamed LM. Access to HIV Care and Resilience in a Long-Term Conflict Setting: A Qualitative Assessment of the Experiences of Living with Diagnosed HIV in Mogadishu, Somali. Int J Environ Res Public Health. 2017;14(7):721. doi: 10.3390/ijerph14070721. PMID: 28678166; PMCID: PMC5551159.
76. Laraqui S, Laraqui O, Manar N, Ghailan T, Belabsir M, Deschamps F, Laraqui CH. The assessment of seafarers' knowledge, attitudes and practices related to STI/HIV/AIDS in northern Morocco. Int Marit Health. 2017;68(1):26-30. doi: 10.5603/IMH.2017.0005. PMID: 28357833.
77. Maatouk I, Nakib ME, Assi M, Farah P, Makso B, Nakib CE, Rady A. Community-led HIV self-testing for men who have sex with men in Lebanon: lessons learned and impact of COVID-19. Health Res Policy Syst. 2021;19(Suppl 1):50. doi: 10.1186/s12961-021-00709-x. PMID: 33882944; PMCID: PMC8058575.
78. Mahfouz AA, Alakija W, al-Khozayem AA, al-Erian RA. Knowledge and attitudes towards AIDS among primary health care physicians in the Asir Region, Saudi Arabia. J R Soc Health. 1995;115(1):23-5. doi: 10.1177/146642409511500108. PMID: 7738977.
79. Memish ZA, Filemban SM, Bamgboyel A, Al Hakeem RF, Elrashied SM, Al-Tawfiq JA. Knowledge and Attitudes of Doctors Toward People Living With HIV/AIDS in Saudi Arabia. J Acquir Immune Defic Syndr. 2015;69(1):61-7. doi: 10.1097/QAI.0000000000000550. PMID: 25642972.
80. Mustafa MA, Mumford SD. Male attitudes towards family planning in Khartoum, Sudan. J Biosoc Sci. 1984;16(4):437-49. doi: 10.1017/s0021932000015273. PMID: 6490682.
81. Naal H, Abboud S, Harfoush O, Mahmoud H. Examining the Attitudes and Behaviors of Health-care Providers Toward LGBT Patients in Lebanon. J Homosex. 2020;67(13):1902-1919. doi: 10.1080/00918369.2019.1616431.
82. Othman SM. Knowledge about HIV/AIDS among high school students in Erbil city/Iraq. Glob J Health Sci. 2014;7(1):16-23. doi: 10.5539/gjhs.v7n1p16. PMID: 25560340; PMCID: PMC4796507.
83. Premadasa G, Sadek M, Ellepola A, Sreedharan J, Muttappallymyalil J. Knowledge of and attitudes towards HIV/AIDS: a survey among dental students in Ajman, UAE. J Investig Clin Dent. 2015;6(2):147-55. doi: 10.1111/jicd.12080. Epub 2013 Dec 20. PMID: 24357612.
84. Rabah DM, Wahdan IH, Merdawy A, Abourafe B, Arafa MA. Oncologists' knowledge and practice towards sperm cryopreservation in Arabic communities. J Cancer Surviv. 2010;4(3):279-83. doi: 10.1007/s11764-010-0140-z. Epub 2010 Jul 23. PMID: 20652434.
85. Salameh P, Zeenny R, Salamé J, Waked M, Barbour B, Zeidan N, Baldi I. Attitudes Towards and Practice of Sexuality Among University Students in Lebanon. J Biosoc Sci. 2016;48(2):233-48. doi: 10.1017/S0021932015000139. Epub 2015 Jun 4. PMID: 26040203.
86. Saleh MA, Al-Ghamdi YS, Al-Yahia OA, Shaqran TM, Mosa AR. Impact of health education program on knowledge about AIDS and hiv transmission in students of secondary schools in buraidah city, saudi arabia: an exploratory study. J Family Community Med. 1999;6(1):15-21. PMID: 23008592; PMCID: PMC3437069.
87. Saleh WF, Gamaleldin SF, Abdelmoty HI, Raslan AN, Fouda UM, Mohesen MN, Youssef MA. Reproductive health and HIV awareness among newly married Egyptian couples without formal education. Int J Gynaecol Obstet. 2014;126(3):209-12. doi: 10.1016/j.ijgo.2014.02.027.
88. Shah S, Elgalib A, Al-Wahaibi A, Al-Fori M, Raju P, Al-Skaiti M, Al-Mashani HN, Duthade K, Omaar I, Muqeetullah M, Mitra N, Shah P, Amin M, Morkos E, Vaidya V, Al-Habsi Z, Al-Abaidani I, Al-Abri SS. Knowledge, Attitudes and Practices Related to HIV Stigma and Discrimination Among Healthcare Workers in Oman. Sultan Qaboos Univ Med J. 2020;20(1):e29-e36. doi: 10.18295/squmj.2020.20.01.005.
89. Spindler E, Bitar N, Solo J, Menstell E, Shattuck D. Jordan's 2002 to 2012 Fertility Stall and Parallel USAID Investments in Family Planning: Lessons From an Assessment to Guide Future Programming. Glob Health Sci Pract. 2017;5(4):617-629. doi: 10.9745/GHSP-D-17-00191. PMID: 29284697; PMCID: PMC5752608.
90. Stycos JM, Sayed HA, Avery R. An evaluation of the population and development program in Egypt. Demography. 1985;22(3):431-43. PMID: 4043454.
91. Valadez JJ, Berendes S, Jeffery C, Thomson J, Ben Othman H, Danon L, Turki AA, Saffialden R, Mirzoyan L. Filling the Knowledge Gap: Measuring HIV Prevalence and Risk Factors among Men Who Have Sex with Men and Female Sex Workers in Tripoli, Libya. PLoS One. 2013;8(6):e66701. doi: 10.1371/journal.pone.0066701. PMID: 23840521; PMCID: PMC3686727.
92. Warren CW, Hiyari F, Wingo PA, Abdel-Aziz AM, Morris L. Fertility and family planning in Jordan: results from the 1985 Jordan Husbands' Fertility Survey. Stud Fam Plann. 1990;21(1):33-9. PMID: 2315966.
93. Sexty RE, Hamadneh J, Rösner S, Strowitzki T, Ditzen B, Toth B, Wischmann T. Cross-cultural comparison of fertility specific quality of life in German, Hungarian and Jordanian couples attending a fertility center. Health Qual Life Outcomes. 2016;14:27. doi: 10.1186/s12955-016-0429-3. PMID: 26911144; PMCID: PMC4765134.
94. Akhu-Zaheya LM, Masadeh AB. Sexual information needs of Arab-Muslim patients with cardiac problems. Eur J Cardiovasc Nurs. 2015;14(6):478-85. doi: 10.1177/1474515115597353.
95. Bdair, I.A., Maribbay, G.L. Perceived Knowledge, Practices, Attitudes and Beliefs of Jordanian Nurses Toward Sexual Health Assessment of Patients with Coronary Artery Diseases. Sex Disabil 38, 491–502 (2020). <https://doi.org/10.1007/s11195-020-09639-y>
96. Sait M, Aljarbou A, Almannie R, Binsaleh S. Knowledge, attitudes, and perception patterns of contraception methods: Cross-sectional study among Saudi males. Urol Ann. 2021;13(3):243-253. doi: 10.4103/UA.UA_42_20.
97. Zaidouni A, Ouasmani F, Benbella A, Ktiri F, Abidli Z, Bezad R. What are the needs of infertile Moroccan couples in Assisted Reproductive Technology?: Exploratory qualitative study in the first fertility public center in Morocco. Bangladesh Journal of Medical Science. 2020;19(4), 697–704. <https://doi.org/10.3329/bjms.v19i4.46628>
98. Barry M, Ghonem L, Albeeshi N, Alrabiah M, Alsharidi A, Al-Omar HA. Resource Utilization and Caring Cost of People Living with Human Immunodeficiency Virus (PLHIV) in Saudi Arabia: A Tertiary Care University Hospital Experience. Healthcare (Basel). 2022;10(1):118. doi: 10.3390/healthcare10010118. PMID: 35052282; PMCID: PMC8776132.
